# Supplementary material for: High-Density and Monodisperse Electrochemical Gold Nanoparticle Synthesis Utilizing the Properties of Boron-Doped Diamond Electrodes
Source: Nanomaterials (Basel). 2022 May 19;12(10):1741. doi: 10.3390/nano12101741 (PMC9144727; doi:10.3390/nano12101741)
Supplement: Supplementary file 1 [file nanomaterials-12-01741-s001.zip › nanomaterials-1716387-supplementary.pdf]

nanomaterials

Supplementary Materials

High-Density and Monodisperse Electrochemical Gold Nanoparticle Synthesis Utilizing the Properties of Boron-Doped Diamond Electrodes

Kenshin Takemura *, Wataru Iwasaki, Nobutomo Morita and Shinya Ohmagari

Sensing System Research Center, The National Institute of Advanced Industrial Science and Technology (AIST), Tosu 841-0052, , Japan; wataru.iwasaki@aist.go.jp (W.I.); morita.nobutomo@aist.go.jp (N.M.); shinya.ohmagari@aist.go.jp (S.O.)

* Correspondence: takemura.kenshin@aist.go.jp; Tel.: +81-942-81-3619

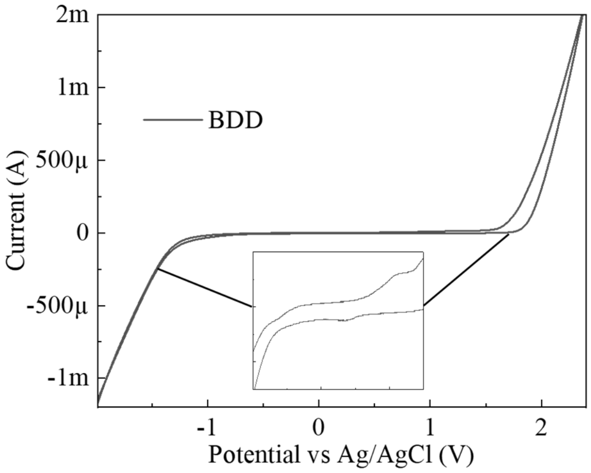

Figure S1. Potential window in AcONa of the BDD measured using cyclic voltammetry. The voltage was swept from –2.0 to 2.4 V with a scan rate 0.2 V/sec.

Table S1. Particle size and number of AuNPs electrodeposited at different durations. The particle analysis was performed using ImageJ.

	Average Particle Size (nm)	Particle Number/μm^2
30 s	42±6	141±3
60 s	56±5	132±7
90 s	120±10	82±7
120 s	90±10	50±10

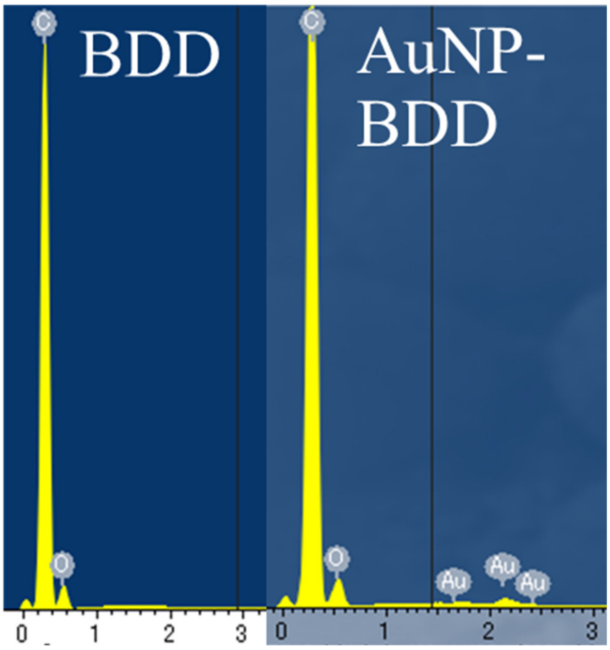

Figure S2. EDX analysis to confirm Au deposition on BDD.

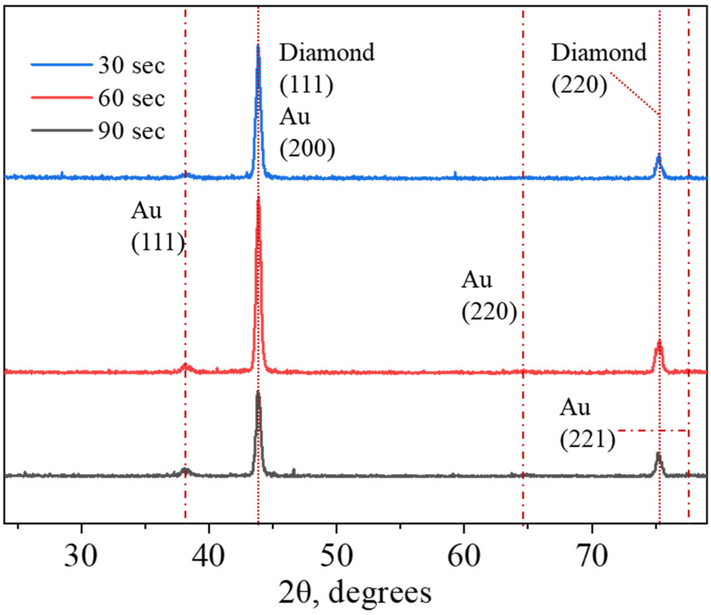

Figure S3. XRD patterns of AuNP-modified BDD samples at different synthesis durations.

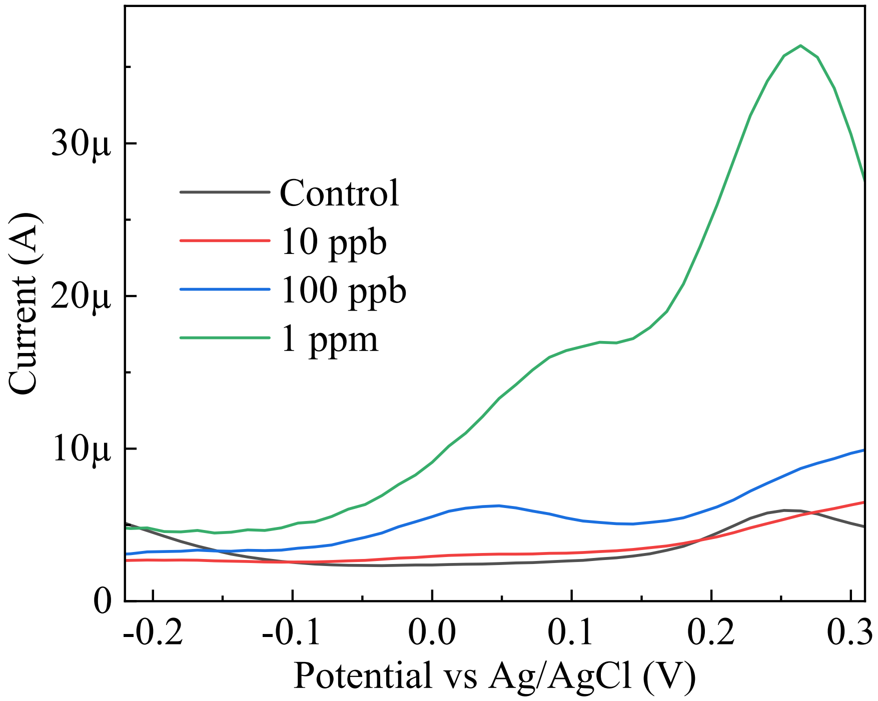

Figure S4. Electrochemical detection of As(III) using BDD in AcONa under the same SWASV condition as with AuNP-BDD.

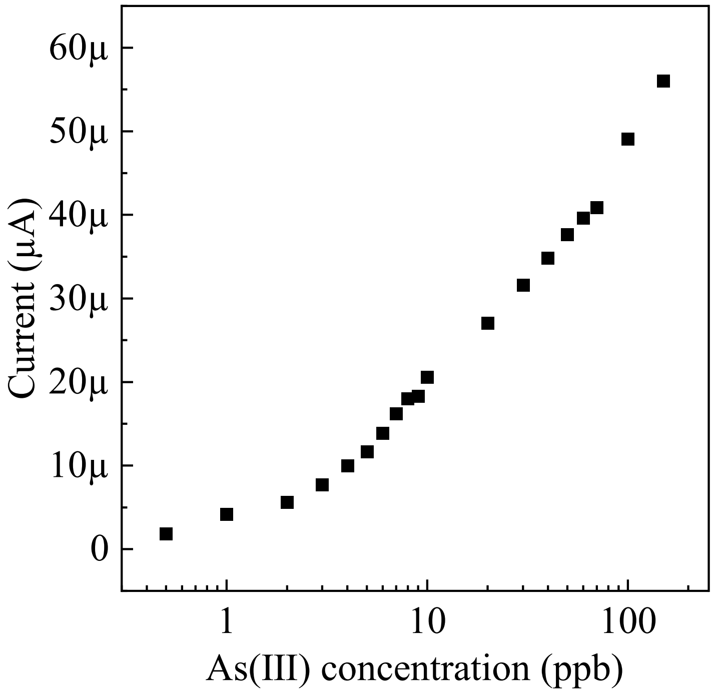

Figure S5. Current peak value plot for the electrochemical detection of As(III) in AcONa.

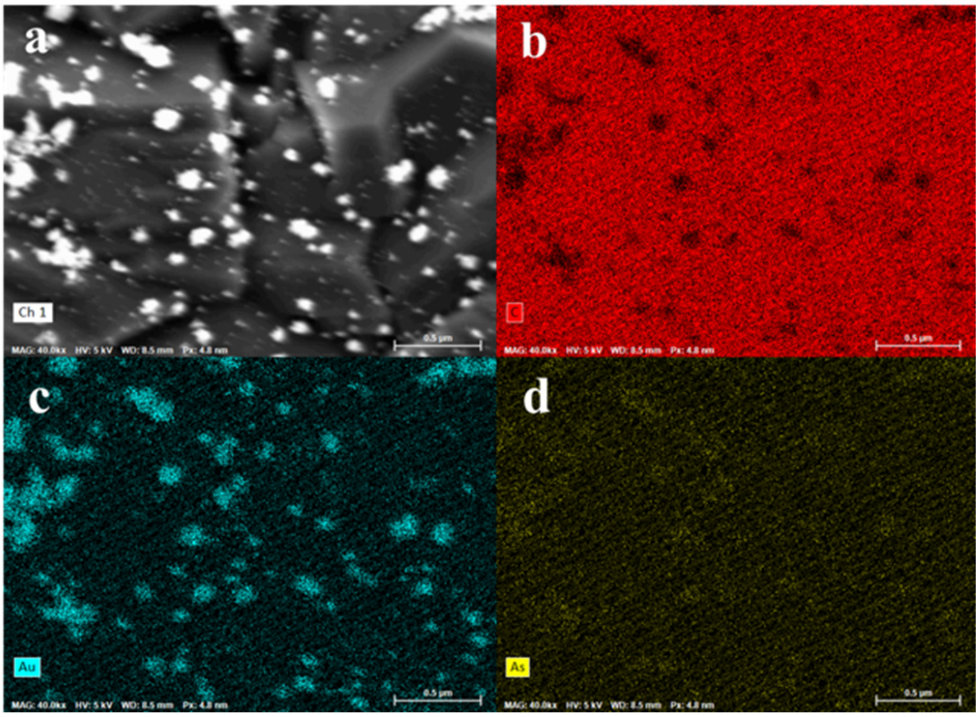

Figure S6. EDX maps of AuNP-BDD after As(III) detection. (**a**) Image obtained in normal SEM mode. (**b–d**) Mapping result of C, Au, and As.
